# Saline versus Plasma Solution-A in Initial Resuscitation of Patients with Out-of-Hospital Cardiac Arrest: A Randomized Clinical Trial

**DOI:** 10.3390/jcm12155040

**Published:** 2023-07-31

**Authors:** Jae-Hyug Woo, Yong Su Lim, Jin Seong Cho, Hyuk Jun Yang, Jae Ho Jang, Jea Yeon Choi, Woo Sung Choi

**Affiliations:** Department of Emergency and Critical Care Medicine, Gachon University Gil Medical Center, Gachon University College of Medicine, Incheon 21565, Republic of Korea; emmetalkiller@gilhospital.com (J.-H.W.);

**Keywords:** cardiopulmonary resuscitation, out-of-hospital cardiac arrest, crystalloid solutions, acidosis, chlorides, saline solution

## Abstract

Background: Although saline is commonly used during cardiopulmonary resuscitation (CPR) or post-cardiac arrest care, it has detrimental effects. This trial aimed to evaluate the efficacy of a balanced crystalloid solution (Plasma Solution-A [PS]) in out-of-hospital cardiac arrest (OHCA) patients and compare it with the efficacy of saline. Methods: A randomized, unblinded clinical trial was conducted using PS and saline for intravenous fluid administration during CPR and post-cardiac arrest care of non-traumatic OHCA patients admitted to the emergency department of a tertiary university hospital. Patients received saline (saline group) or PS (PS group) within 24 h of hospital arrival. The primary outcomes were changes in arterial pH, bicarbonate, base excess (BE), and chloride levels within 24 h. The secondary outcomes were clinical outcomes including mortality. Results: Of the 364 patients, data from 27 and 26 patients in the saline and PS groups, respectively, were analyzed. Analysis using a linear mixed model revealed a significant difference in BE change over time between the groups (treatment-by-time *p* = 0.044). Increase in BE and bicarbonate levels from 30 min to 2 h was significantly greater (*p* = 0.044 and *p* = 0.024, respectively) and the incidence of hyperchloremia was lower (*p* < 0.001) in the PS group than in the saline group. However, there was no difference in clinical outcomes. Conclusion: Use of PS for resuscitation resulted in a faster improvement in BE and bicarbonate, especially in the early phase of post-cardiac arrest care, and lower hyperchloremia incidence than the use of saline, without differences in clinical outcomes, in OHCA patients.

## 1. Introduction

Although the optimal intravenous (IV) fluid for resuscitation remains undetermined, saline is the most commonly used crystalloid worldwide [1]. The chloride (Cl^−^) concentration of 0.9% saline is higher than that of human plasma, and therefore can potentially contribute to the development of hyperchloremic metabolic acidosis and thereby lead to immune and renal dysfunction [2,3,4]. To avoid these undesirable side effects, studies on the use of balanced crystalloids in critically ill patients have emerged. Several studies have reported that the use of balanced crystalloids as the initial fluid resuscitation for patients with diabetic ketoacidosis or traumatic injuries has resulted in better acid–base balance and renal outcomes [5,6]. Based on these findings, the use of balanced crystalloids in the intensive care unit (ICU) has increased [7]. However, recent studies on critically ill adults have shown conflicting results regarding the effects of balanced crystalloids on death and renal outcome [8,9]. To the best of our knowledge, however, there have been no studies on the use of balanced crystalloids during cardiopulmonary resuscitation (CPR) of out-of-hospital cardiac arrest (OHCA) patients in the emergency department (ED) or during the treatment of post-cardiac arrest syndrome (PCAS) in the ICU [10].

Saline is a commonly used fluid during CPR or post-cardiac arrest care. However, whether saline is the optimal IV fluid to use during these interventions remains unclear. Saline causes metabolic acidosis, which causes arrhythmias; these two interrelated pathological changes may lead to cardiac arrest [11,12]. Accordingly, saline-induced acidosis could interfere with the recovery of OHCA patients and deprive them of stable post-cardiac arrest care. Additionally, immune or renal dysfunction caused by high doses of saline may lead to death in patients with PCAS. Therefore, it is necessary to identify a more optimal resuscitation fluid than saline for the treatment of patients with cardiac arrest.

Balanced crystalloids could be a more optimal resuscitation fluid during the treatment of OHCA patients because they contain buffers and have lower Cl^−^ concentrations. Therefore, we compared a balanced crystalloid with saline in this randomized trial conducted among patients with OHCA. We hypothesized that using a balanced crystalloid would result in better improvements of acid–base and electrolyte balance, similar effects on hemodynamic stability, and higher survival rates than when using saline.

## 2. Materials and Methods

We conducted an investigator-initiated, pragmatic, randomized, unblinded, parallel-group clinical trial in which the use of a balanced crystalloid (Plasma Solution-A [PS]) was compared with the use of saline in IV fluid administration during CPR and post-cardiac arrest care of non-traumatic OHCA patients admitted to a tertiary university hospital ED between 1 April 2019, and 15 July 2020 (clinical trial number KCT0003587, https://cris.nih.go.kr/cris/index.jsp, accessed on 6 March 2019). PS (HK inno.N Corp., Seoul, Republic of Korea) contains the same ingredients as Plasma-Lyte A (Supplementary File S1: Study protocol). No changes were made to the trial protocol after the trial commenced. The study protocol was approved by the Gachon University Gil Medical Center IRB (GCIRB2017-293). The IRB permitted an initial waiver of consent because it was not practical to obtain informed consent before initiating IV administration. We attempted to obtain consent from the surrogate as soon as possible after the return of spontaneous circulation (ROSC). This trial complied with the principles of the Declaration of Helsinki. This manuscript follows the Consolidated Standards of Reporting Trials (CONSORT) guidelines.

### 2.1. Randomization

Participants were randomized 1:1 to receive either PS or saline for any IV administration of isotonic crystalloids. Randomization was achieved using permuted block randomization with a block size of 4. An author developed a randomization list using a web-based randomization program to generate trial IDs and treatment allocations before starting the trial. Blinding was not implemented.

### 2.2. Trial Design and Intervention

Details including the inclusion and exclusion criteria, are described in Supplementary File S1: Study protocol. We consecutively screened patients aged ≥18 years who were admitted to our ED after OHCA irrespective of their initial cardiac rhythm. Participants were randomized 1:1 to receive either PS (PS group) or saline (0.9% sodium chloride; saline group). 

The trial interventions were performed for 24 h after the patient visited the ED. Upon arrival at the ED, all patients received only the allocated crystalloid while receiving advanced cardiovascular life support (ACLS). We administered only the allocated crystalloid to the patient for the first 24 h in the hospital. These interventions were continued in the ICU and ED.

Upon arrival at the ED, blood samples were collected to determine serum electrolyte levels and perform a complete blood count, coagulation tests, liver and renal function tests, and arterial blood gas analysis (ABGA). Blood tests were performed repeatedly during the first 24 h according to the protocol. These interventions were discontinued in patients who could not adhere to the trial protocol.

We obtained information concerning patient demographics, characteristics of cardiac arrest, and amount and type of crystalloid administered in prehospital settings. We recorded in-hospital treatments, including the amount and type of IV fluids, blood products, and drugs administered for the first 24 h. We collected laboratory results and clinical data. We monitored the patient′s status for up to 6 months and recorded the neurologic and survival outcomes. The definitions of acute kidney injury (AKI), major adverse kidney events within 30 days (MAKE30), cumulative vasopressor index (CVI), and cerebral performance category (CPC) are in Supplementary File S1: Study protocol [8,13,14,15,16].

### 2.3. Outcomes

The primary outcomes measured were changes in arterial pH, base excess (BE: primary endpoint), and bicarbonate (HCO_3_^−^) and Cl^−^ levels within the first 24 h. The secondary outcomes were clinical outcomes, specifically AKI development within 72 h, MAKE30, stability of hemodynamic status within 24 h, and neurologic and survival outcomes 6 months after OHCA (secondary endpoint: survival to hospital discharge). 

### 2.4. Statistical Analyses

Sample size was calculated based on a previous randomized trial comparing the effect of a balanced crystalloid and saline on BE, as described in Supplementary File S1: Study protocol [6]. At least 24 participants were required in each group, resulting in 48 participants in total.

Data were analyzed using SPSS version 17.0 (SPSS Inc., Chicago, IL, USA) and R version 4.1.0 (R Foundation for Statistical Computing, Vienna, Austria). Continuous variables are reported as medians and interquartile ranges or means and standard deviations, as appropriate. Comparisons were performed using the Mann–Whitney U test or *t*-test. Categorical variables are expressed as frequencies and percentages, and comparisons were performed using the chi-square test or Fisher′s exact test.

We compared the changes in arterial pH, BE, HCO_3_^−^, and Cl^−^ levels over time between the allocated crystalloid groups using linear mixed models (LMM) with fixed effects (each allocated crystalloid, time, and interaction between each allocated crystalloid and time) and random intercepts (study patients). All statistical tests were two-sided, and a *p*-value of <0.05 was considered statistically significant. The *p*-values of arterial pH, BE, HCO_3_^−^, and Cl^−^ levels were corrected using the Benjamini–Hochberg procedure because multiple comparisons were conducted.

## 3. Results

### 3.1. Baseline Characteristics 

**Figure 1:**
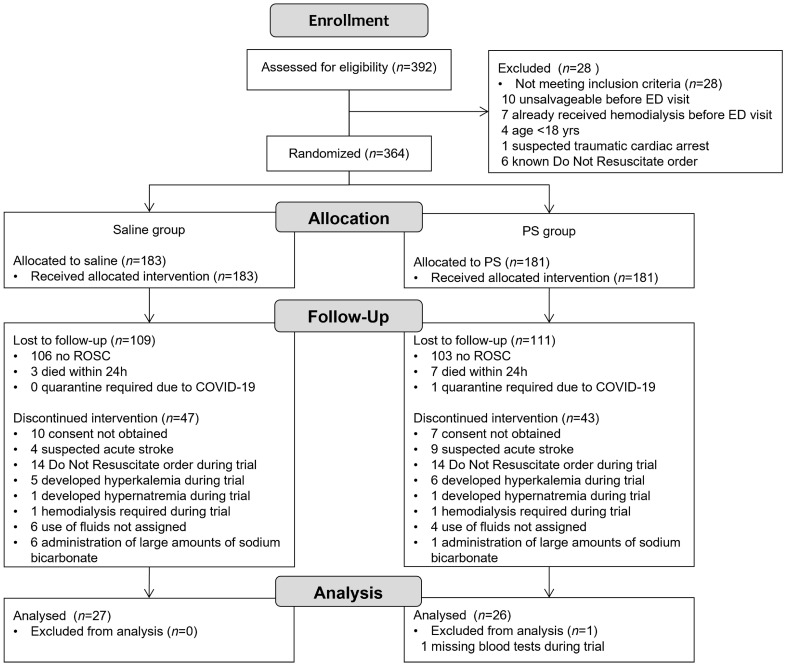
CONSORT flow diagram of the trial. Abbreviations: ED, emergency department; PS, Plasma Solution-A; ROSC, return of spontaneous circulation; COVID-19, coronavirus disease 2019.

During the trial period, of the 392 potential patients with OHCA, 364 were randomized (183 in the saline group and 181 in the PS group) (Figure 1: CONSORT diagram). The proportion of ROSC cases was 42.1% and 43.1% in the saline and PS groups, respectively (Supplementary File S1: Table S1). After excluding patients who could not adhere to the trial protocol, 27 and 26 patients in the saline and PS groups, respectively, were included in the final analysis. 

The baseline characteristics of the patients were not significantly different between the groups (Table 1). In the saline and PS groups, patients had a mean age of 59.0 ± 15.3 and 61.7 ± 14.9 years, and 16 (59.3%) and 18 (69.2%) patients were male, respectively. In each group (saline vs. PS), 13 (48.1%) and 15 (57.7%) patients received ACLS in prehospital settings and 12 (44.4%) and 12 (46.2%) received fluids in prehospital settings (*p* = 0.901), respectively. Only saline was administered in prehospital settings, and the mean volume administered in the saline and PS groups was 0.0 mL (0.0, 400.0) and 0.0 mL (0.0, 300.0), respectively (*p* = 0.961).

### 3.2. Fluid Administration and In-Hospital Treatments after Randomization

Within the first 24 h, the cumulative volume of the allocated crystalloid administered in the saline and PS groups was 4029.0 mL (3562.0, 5650.0) and 3917.5 mL (3640.0, 5420.0) (*p* = 0.742), respectively (Table 1). The number of patients who received diuretics was 5 (18.5%) and 3 (11.5%) in the saline and PS groups (*p* = 0.704), respectively. No patient received sodium bicarbonate in either group. Details of in-hospital treatments are described in Table S2.

### 3.3. Primary Outcomes (Laboratory Results)

**Figure 2:**
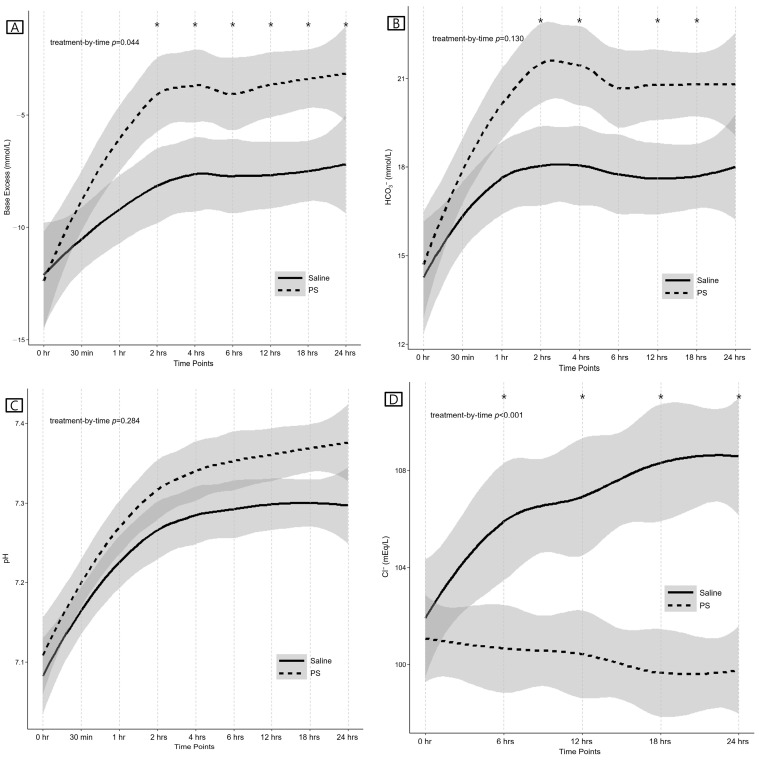
First 24 h trends for (**A**) arterial base excess, (**B**) arterial bicarbonate levels, (**C**) arterial pH, and (**D**) serum chloride levels. Results are reported as the mean and 95% confidence interval (denoted by gray shading). *p*-values in each figure are treatment-by-time *p*-values calculated using the linear mixed model analysis for each outcome repeatedly measured. * indicates a time point with a significant *p*-value corrected using the Benjamini–Hochberg procedure after univariable analysis. Abbreviations: PS, Plasma Solution-A; HCO_3_^−^, bicarbonate; Cl^−^, chloride.

The laboratory results of the two groups are summarized in Table 2, Tables S3 and S4, and Figure 2. At baseline, arterial pH, BE, HCO_3_^−^, and serum Cl^−^ levels were not different between the groups. BE was higher at 2, 4, 6, 12, 18, and 24 h in the PS group than in the saline group (*p* = 0.032, *p* = 0.036, *p* = 0.032, *p* = 0.009, *p* = 0.009, and *p* = 0.036, respectively). The increase in BE within the first 24 h was greater in the PS group than in the saline group (*p* = 0.025). Additionally, the increase in BE from 30 min to 2 h was greater in the PS group than in the saline group (*p* = 0.044). In the LMM analysis, the change in BE was significantly different between the groups over time (treatment-by-time *p* = 0.044) (Figure 2A).

Arterial HCO_3_^−^ levels at 2, 4, 12, and 18 h were higher in the PS group than in the saline group (*p* = 0.034, *p* = 0.034, *p* = 0.034, and *p* = 0.034, respectively). The increase in arterial HCO_3_^−^ level within the first 24 h was greater in the PS group than in the saline group (*p* = 0.036). Additionally, the increase in HCO_3_^−^ from 30 min to 2 h was greater in the PS group than in the saline group (*p* = 0.024). In the LMM analysis, the change in arterial HCO_3_^−^ level did not differ between the groups over time (treatment-by-time *p* = 0.130) (Figure 2B). 

The arterial pH at each time point was higher in the PS group than in the saline group, although not significantly so. In the LMM analysis, the change in arterial pH did not differ between the groups over time (treatment-by-time *p* = 0.284) (Figure 2C). 

The serum Cl^−^ levels at 6, 12, 18, and 24 h were lower in the PS group than in the saline group (*p* = 0.003, *p* < 0.001, *p* < 0.001, and *p* < 0.001, respectively). The change in serum Cl^−^ level within the first 24 h was smaller in the PS group than in the saline group (*p* < 0.001). In the LMM analysis, the change in serum Cl^−^ level was significantly different between the groups over time (treatment-by-time *p* < 0.001) (Figure 2D). Increases in serum Cl^−^ level and the development of hyperchloremia within 24 h were more frequent in the saline group than in the PS group (both *p* < 0.001). At every time point, the development of hyperchloremia was more frequent in the saline group than in the PS group.

### 3.4. Secondary Outcomes (Clinical Outcomes)

Clinical outcomes are summarized in Table 3 and Table S5. Blood pressure, heart rate, and CVI did not differ at any time point. Total urine output within 24 h was 3220.5 ± 1919.0 mL and 3686.8 ± 2235.2 mL in the saline and PS groups, respectively (*p* = 0.418). AKI development within 72 h and MAKE30 did not differ between the groups (*p* = 0.288 and *p* = 0.064, respectively). Survival and a good CPC at 6 months also did not differ between the groups (*p* = 0.318 and *p* = 0.328, respectively).

### 3.5. Sensitivity Analyses

The sensitivity analyses relating to the primary and secondary endpoints are described in Supplementary File S1: Sensitivity analyses. Absolute differences of the primary endpoint (the increase in BE within the first 24 h) before and after adjustment across the groups were 5.2 mmol/L (95% CI: 1.2–9.2, unadjusted) and 5.3 mmol/L (95% CI: 1.2–9.4, adjusted). The adjusted odds ratios for the secondary endpoint (survival to hospital discharge) were not significant at each discontinuation point of the trial.

## 4. Discussion

To the best of our knowledge, no randomized studies have evaluated the efficacy of balanced crystalloids in the treatment of OHCA patients. In this trial investigating PS, a balanced crystalloid, although there was no significant difference in the improvement of arterial pH between the saline and PS groups, BE and HCO_3_^−^ levels improved more, and at a faster rate, in the PS group. The development of hyperchloremia was less frequent in the PS group than in the saline group. Hemodynamic stability, AKI development, and survival rates did not differ between the groups.

Balanced crystalloids contain near-physiological amounts of Cl^−^, and anions such as lactate, acetate, and gluconate, which act as buffers to generate bicarbonate in the human body [17]. They are relatively hypotonic, because they have lower sodium concentrations than the extracellular environment [1]. Excessive administration of balanced crystalloids may result in metabolic alkalosis, hyperlactatemia, hypotonicity (due to sodium lactate), and cardiotoxicity (due to acetate) [1,18]. However, compared to saline use, the use of balanced crystalloids is associated with a lower incidence of postoperative infection, renal replacement therapy, blood transfusion, and acidosis-related problems [19]. However, there is an ongoing debate regarding the usefulness of this.

In contrast to balanced crystalloids, saline leads to acidosis [2]. Despite this undesirable and serious side effect, saline has been both traditionally and frequently used during CPR and post-cardiac arrest care in prehospital or in-hospital settings. This critical acidosis is not only one of the causes of cardiac arrest, but can also lead to arrhythmias, interfering with the recovery of OHCA patients [11,12]. As acidosis tends to reduce cardiac contractility, it can lead to an unstable hemodynamic status even after ROSC [20]. On the other hand, targeted temperature management (TTM) is performed after ROSC, and one study suggested that TTM with a target temperature of 32–34 °C has adverse effects on patients with OHCA [21]. Lowered body temperature during TTM induces microcirculatory impairment, which subsequently causes lactic acidosis [22]. Lactic acidosis is associated with unfavorable outcomes of patients with OHCA [23]. Since the delayed improvement of acidosis worsens the patient′s condition, it can be difficult to provide stable post-cardiac arrest care. Accordingly, the rapid improvement of acidosis can aid recovery from cardiac arrest and provide an opportunity for stable post-cardiac arrest care for OHCA patients.

In this trial, the mean arterial pH tended to be higher in the PS group than in the saline group at all time points, although there was no significant difference in the degree of arterial pH improvement between the groups over time, contrary to expectations. This might be due to the small number of cases in this trial. Most previous studies comparing balanced crystalloids with saline also showed no significant difference in arterial pH between groups but did show significant differences in BE or HCO_3_^−^ levels [5,24,25]. On the other hand, more patients discontinued participation in the trial owing to the administration of high-dose sodium bicarbonate in the saline group than in the PS group. In these patients, sodium bicarbonate was administered because the patient′s clinical course worsened because of an insufficient improvement in acidosis, despite ROSC. Owing to these discontinuation events, a bias might have been introduced. In addition, the patients were intubated and mechanically ventilated. The status of ventilation support in each patient could also have influenced the pH or carbon dioxide levels that affected the pH. Unlike pH, BE showed a significant difference between the groups at every time point, starting 2 h after the initiation of the trial. The BE and arterial HCO_3_^−^ levels improved notably faster between 30 min and 2 h in the PS group than in the saline group. This suggests that the administration of balanced crystalloids might be helpful for the initial stabilization of an OHCA patient’s condition; this period is when post-cardiac arrest care after ROSC should be started. After ROSC, post-cardiac arrest care can be attempted more efficiently if the patient’s condition is stable. Accordingly, the use of balanced crystalloids might be helpful in the treatment of patients with OHCA. Studies with a larger number of cases are needed to build more robust evidence supporting this.

Saline also leads to hyperchloremia, and saline-induced hyperchloremic acidosis induces immune or renal dysfunction and reductions in arterial blood pressure [2,3,4,26,27]. A recent report suggests that hyperchloremia is associated with poor neurologic outcomes in OHCA patients and that a chloride-restricted solution could be preferable at the early stage after cardiac arrest [28]. A decrease in blood pressure could interfere with stable post-cardiac arrest care and may reduce cerebral blood flow, leading to poor neurologic outcomes [29]. The use of fluids containing high Cl^−^ concentrations may cause immune or renal dysfunction, which may lead to the death of patients with PCAS [4,27]. In this trial, hyperchloremia never developed in the PS group, and the Cl^−^ elevation was less frequent in the PS group than in the saline group. In terms of hemodynamic status, it can be deduced that the two crystalloids showed similar hemodynamic effects, as there were no differences in the blood pressure, heart rate, and CVI between the groups, despite their having received similar amounts of the allocated crystalloid. The incidence of immune and renal complications did not differ between the groups. However, in our trial, as we did not examine cytokines in detail and only checked the white blood cell count and C-reactive protein level, the assessment of changes in the immune system was uncertain. The incidence of AKI did not appear to differ between the groups because of the small number of cases in this trial. Recent studies in critically ill adults have shown conflicting results regarding the effects of balanced crystalloids on renal outcome [8,9]. The assessment of AKI development in OHCA patients remains uncertain, warranting further studies. However, as hyperchloremia—which causes various problems—occurs less frequently, balanced crystalloids may be more useful.

Considering the advantages described above, balanced crystalloids might be more optimal as resuscitative fluids; however, they have some disadvantages. First, some balanced crystalloids contain potassium, while some OHCA patients have hyperkalemia that causes cardiac arrest [12,17]. In this trial, we inevitably discontinued the study for patients with the following hyperkalemia-related conditions: hyperkalemia found in the initial ABGA, initial pseudohyperkalemia owing to hemolysis caused by the difficulty of sampling, and cases where the patients were already on dialysis. Accordingly, the use of balanced crystalloids is limited in cardiac arrest patients with suspected hyperkalemia. Second, the occurrence of a hypoosmolar state should be noted. Although the clinical significance of this problem needs to be studied further, we found that the measured and calculated osmolality was slightly lower at 24 h in the PS group than in the saline group. Because low osmolality may worsen the cerebral edema that often occurs in patients with PCAS, caution is required [30]. If a resuscitation fluid overcoming these disadvantages can be developed, it can be used more widely in OHCA patients.

This study has some limitations. First, because this was a single-center study with a small study sample, it is difficult to generalize these results. Furthermore, the sample size in this trial could be insufficient for assessing secondary outcomes; thus, additional large-scale studies are needed. Second, the assessment of some outcomes may be limited given that this was not a blinded trial. Third, as acidosis in the initial phase of the clinical course in post-cardiac arrest patients is common and critical, we observed patients’ laboratory results only during the initial 24 h; thus, it remains unclear whether the groups differed at time points after 24 h. Fourth, the final dropout rate exceeded that expected by the authors; the cause of discontinued intervention, and thus the number of dropout cases, could not be precisely predicted during the trial planning stage. Fifth, bias might have been introduced because the trial was discontinued in many patients owing to the absence of ROSC or death within 24 h (an inevitable limitation of a trial on OHCA patients). Sixth, because the participation of patients receiving high-dose sodium bicarbonate during the trial was discontinued, those with more severe acidosis might have been excluded from this trial. Seventh, ventilation support was not specifically controlled in this trial. Ventilation support was provided to the patient according to our institution′s protocol, which was written according to a previous study (target SpO2, 94–98%; target PaCO2, 35–45 mmHg) [31]. According to previous studies, both hypoxemia and hyperoxemia are associated with unfavorable outcomes of patients with OHCA [32,33]. This uncontrolled effect might have influenced the secondary outcomes. Eighth, it is important to note that some primary outcomes (such as BE and HCO_3_^−^ levels) are calculated after the measurement of carbon dioxide levels and pH in ABGA. This calculation may have resulted in these primary outcomes affecting each other′s results. Lastly, we assessed fluid responsiveness using serial central venous pressure and blood pressure monitoring and the respiratory variability of the inferior vena cava according to the physician′s preference. In addition, if available, a machine was used to monitor the fluid responsiveness. However, this study did not specifically control for these monitoring methods. Numerous methods, such as pulse pressure variation, passive leg raising, and stroke volume variation, were recently studied [34,35,36]. In future studies, it will be necessary to strictly assess fluid responsiveness using these methods and to administer crystalloids.

## 5. Conclusions

In OHCA patients, resuscitation using PS (a balanced crystalloid) resulted in a faster improvement in BE and HCO_3_^−^ levels than resuscitation using saline, especially in the early phase of post-cardiac arrest care. Hyperchloremia occurred less frequently in patients who were administered PS than in those who were administered saline. Hemodynamic stability, AKI development, and survival did not differ between the saline and PS groups. Additional large-scale randomized studies are required.

## Figures and Tables

**Table 1:** Baseline characteristics of patients receiving saline versus Plasma Solution-A.

Variables	Saline Group (*n* = 27)	PS Group (*n* = 26)	*p*-Value
Age (years)	59.0 ± 15.3	61.7 ± 14.9	0.526
Sex (male), *n* (%)	16 (59.3)	18 (69.2)	0.449
Location of arrest (residence), *n* (%)	13 (48.1)	15 (57.7)	0.487
Bystander CPR, *n* (%)	13 (48.1)	15 (57.7)	0.593
Cardiac etiology, *n* (%)	18 (66.7)	19 (73.1)	0.899
Initial rhythm (shockable), *n* (%)	13 (48.1)	17 (65.4)	0.206
Interval from collapse to BLS (min)	5.1 ± 4.5	4.9 ± 4.8	0.862
Interval from BLS to ROSC (min)	14.0 (9.0, 21.5)	14.0 (9.0, 21.0)	0.845
Interval from collapse to ED arrival (min)	26.9 ± 12.6	30.3 ± 11.7	0.317
ROSC before ED arrival (yes), *n* (%)	17 (63.0)	21 (80.8)	0.150
Pre-existing illness, *n* (%)			
Diabetes mellitus	9 (33.3)	6 (23.1)	0.407
Hypertension	17 (63.0)	17 (65.4)	0.854
CVA	4 (14.8)	1 (3.8)	0.351
Cardiac disease	10 (37.0)	7 (26.9)	0.430
Cancer	1 (3.7)	1 (3.8)	1.000
ACLS in prehospital settings (yes), *n* (%)	13 (48.1)	15 (57.7)	0.487
Fluid administration in prehospital settings (yes), *n* (%)	12 (44.4)	12 (46.2)	0.901
Fluids administered in prehospital settings (saline), *n* (%)	12 (44.4)	12 (46.2)	0.901
Volume of fluid administered in prehospital settings (mL)	0.0 (0.0, 400.0)	0.0 (0.0, 300.0)	0.961
In-hospital treatments within 24 h			
Cumulative volume of the allocated crystalloid administered (mL)	4029.0 (3562.0, 5650.0)	3917.5 (3640.0, 5420.0)	0.742
Administration of diuretics (yes), *n* (%)	5 (18.5)	3 (11.5)	0.704
Administration of insulin (yes), *n* (%)	11 (40.7)	9 (34.6)	0.646
Administration of sodium bicarbonate (yes), *n* (%)	0 (0.0)	0 (0.0)	N/C
Intravenous potassium replacement (yes), *n* (%)	3 (11.1)	1 (3.8)	0.610
Intravenous calcium replacement (yes), *n* (%)	3 (11.1)	2 (7.7)	1.000
Intravenous magnesium replacement (yes), *n* (%)	3 (11.1)	1 (3.8)	0.610
Intravenous phosphate replacement (yes), *n* (%)	1 (3.7)	0 (0.0)	1.000
Transfusion (yes), *n* (%)	2 (7.4)	1 (3.8)	1.000
Targeted temperature management, *n* (%)	24 (88.9)	24 (92.3)	1.000

Values are expressed as numbers (percentages), medians (interquartile ranges), or means ± standard deviations. Details of the in-hospital treatments are described in Supplementary Materials (Supplementary File S1). Abbreviations: PS, Plasma Solution-A; CPR, cardiopulmonary resuscitation; BLS, basic life support; ROSC, return of spontaneous circulation; ED, emergency department; CVA, cerebrovascular attack; ACLS, advanced cardiovascular life support; N/C, not calculated.

**Table 2:** Laboratory outcomes of patients receiving saline versus Plasma Solution-A.

Variables	Saline Group (*n* = 27)	PS Group (*n* = 26)	Absolute Difference (95% CI) †	*p*-Value
Base excess				
at baseline (mmol/L)	−14.9 (−16.3, −9.3)	−12.8 (−15.4, −8.8)	−0.1 (−4.5 to 4.3)	0.595 *
at 6 h (mmol/L)	−7.9 ± 5.5	−4.1 ± 4.9	3.7 (0.8 to 6.6)	0.032 *
at 24 h (mmol/L)	−7.2 ± 6.1	−3.4 ± 5.5	3.9 (0.6 to 7.1)	0.036 *
Increase in base excess (0–2 h, mmol/L)	3.9 ± 7.3	8.4 ± 4.6	4.5 (0.7 to 8.3)	0.021
Increase in base excess (0–24 h, mmol/L)	5.5 (1.4, 8.7)	9.4 (5.9, 12.2)	5.2 (1.2 to 9.2)	0.025
Arterial HCO_3_^−^				
at baseline (mmol/L)	14.1 ± 4.5	14.8 ± 5.4	0.7 (−2.2 to 3.7)	0.625 *
at 6 h (mmol/L)	17.8 ± 5.1	20.5 ± 4.1	2.7 (0.1 to 5.3)	0.062 *
at 24 h (mmol/L)	18.8 (15.0, 20.1)	20.2 (17.7, 22.8)	2.5 (−0.2 to 5.3)	0.062 *
Increase in arterial HCO_3_^−^ (0–2 h, mmol/L)	4.0 ± 3.8	6.8 ± 3.5	2.8 (0.6 to 5.0)	0.015
Increase in arterial HCO_3_^−^ (0–24 h, mmol/L)	2.8 ± 5.1	5.9 ± 4.3	3.0 (0.2 to 5.8)	0.036
Arterial pH				
at baseline	7.08 ± 0.19	7.11 ± 0.18	0.03 (−0.08 to 0.13)	0.620 *
at 6 h	7.29 ± 0.11	7.35 ± 0.12	0.07 (0.00 to 0.13)	0.108 *
at 24 h	7.30 ± 0.11	7.38 ± 0.11	0.08 (0.02 to 0.14)	0.099 *
Increase in arterial pH (0–2 h)	0.18 ± 0.15	0.20 ± 0.18	0.02 (−0.08 to 0.11)	0.718
Increase in arterial pH (0–24 h)	0.20 ± 0.17	0.27 ± 0.15	0.06 (−0.03 to 0.16)	0.169
Serum chloride				
at baseline (mEq/L)	101.9 ± 6.3	101.1 ± 5.1	−0.8 (−4.0 to 2.3)	0.593 *
at 6 h (mEq/L)	105.9 ± 6.3	100.7 ± 5.0	−5.2 (−8.4 to −2.1)	0.003 *
at 24 h (mEq/L)	108.6 ± 5.7	99.8 ± 4.8	−8.8 (−11.7 to −5.9)	<0.001 *
Change in serum chloride (0–24 h, mEq/L)	7.0 (2.0, 9.5)	−1.0 (−3.0, 0.0)	−8.0 (−10.3 to −5.6)	<0.001
Increase in serum chloride (0–24 h, yes), *n* (%)	25 (92.6)	5 (19.2)	−73.4 (−91.5 to −55.3)	<0.001
Development of hyperchloremia within 24 h (yes), *n* (%)	13 (48.1)	0 (0.0)	N/C	<0.001
Development of hypernatremia within 24 h (yes), *n* (%)	1 (3.7)	0 (0.0)	N/C	1.000
Development of hyperosmolar state within 24 h (yes), *n* (%)	4 (14.8)	4 (15.4)	0.6 (−18.7 to 19.9)	1.000
Development of hypoosmolar state within 24 h (yes), *n* (%)	16 (59.3)	19 (73.1)	13.8 (−11.4 to 39.0)	0.288
Serum creatinine				
at baseline (mg/dL)	1.2 (0.9, 1.3)	1.1 (0.9, 1.3)	−0.2 (−0.4 to 0.0)	0.434
at 24 h (mg/dL)	0.7 (0.6, 1.0)	0.7 (0.5, 0.9)	−0.2 (−0.6 to 0.1)	0.413
Change in serum creatinine (0–24 h, mg/dL)	−0.4 (−0.5, −0.2)	−0.4 (−0.5, −0.2)	−0.1 (−0.3 to 0.2)	0.859

Values are expressed as numbers (percentages), medians (interquartile ranges), or means ± standard deviations. The details of the laboratory results are described in Supplementary Materials (Supplementary File S1). * indicates corrected *p*-values using the Benjamini–Hochberg procedure because multiple comparisons were conducted. † The differences shown are expressed as percentage points or mean differences. Abbreviations: PS, Plasma Solution-A; CI, confidence interval; HCO_3_^−^, bicarbonate; hyperchloremia, serum chloride level > 110 mEq/L; N/C, not calculated; hypernatremia, serum sodium level > 145 mEq/L; hyperosmolar state, serum osmolality > 308 mOsm/kg; hypoosmolar state, serum osmolality < 289 mOsm/kg.

**Table 3:** Clinical outcomes of patients receiving saline versus Plasma Solution-A.

Variables	Saline Group (*n* = 27)	PS Group (*n* = 26)	Absolute Difference (95% CI) †	*p*-Value
SBP at ICU admission (mmHg)	125.9 ± 40.9	126.9 ± 46.8	1.0 (−23.2 to 25.2)	0.934
at 6 h after ED visit (mmHg)	110.1 ± 28.0	123.9 ± 34.4	13.8 (−3.5 to 31.0)	0.115
at 12 h after ED visit (mmHg)	121.3 ± 29.1	118.4 ± 29.4	−2.9 (−19.0 to 13.2)	0.722
at 24 h after ED visit (mmHg)	119.6 ± 21.7	115.2 ± 27.0	−4.4 (−17.9 to 9.1)	0.512
Highest SBP within 24 h (mmHg)	171.0 (157.5, 203.5)	173.5 (151.0, 196.0)	−6.4 (−25.5 to 12.6)	0.702
Lowest SBP within 24 h (mmHg)	71.4 ± 15.4	75.3 ± 15.0	3.9 (−4.5 to 12.3)	0.355
HR at ICU admission (per min)	91.1 ± 25.8	89.5 ± 23.6	−1.6 (−15.3 to 12.1)	0.814
at 6 h after ED visit (per min)	79.7 ± 29.7	83.5 ± 24.5	3.8 (−11.3 to 18.8)	0.615
at 12 h after ED visit (per min)	74.0 ± 24.9	77.2 ± 22.2	3.2 (−9.8 to 16.2)	0.625
at 24 h after ED visit (per min)	70.0 (58.0, 85.0)	70.0 (62.0, 80.0)	−4.1 (−16.2 to 7.9)	0.624
Highest HR within 24 h (per min)	115.3 ± 24.1	125.8 ± 27.1	10.5 (−3.6 to 24.6)	0.142
Lowest HR within 24 h (per min)	60.0 (45.5, 80.0)	53.0 (46.0, 66.0)	−3.3 (−16.1 to 9.5)	0.682
CVI at ICU admission	0.0 (0.0, 3.0)	0.0 (0.0, 4.0)	1.0 (−0.4 to 2.5)	0.274
at 6 h after ED visit	0.0 (0.0, 4.0)	2.0 (0.0, 4.0)	0.3 (−1.0 to 1.6)	0.644
at 12 h after ED visit	2.0 (0.0, 4.0)	2.0 (0.0, 4.0)	0.4 (−1.4 to 2.2)	0.904
at 24 h after ED visit	2.0 (0.0, 3.5)	1.5 (0.0, 4.0)	0.0 (−1.9 to 1.8)	0.661
Total urine output within 24 h (mL)	3220.5 ± 1919.0	3686.8 ± 2235.2	466.3 (−681.1 to 1613.7)	0.418
Development of AKI within 72 h, *n* (%)	11 (40.7)	7 (26.9)	−13.8 (−39.0 to 11.4)	0.288
Development of MAKE30, *n* (%)	14 (51.9)	7 (26.9)	−24.9 (−50.3 to 0.5)	0.064
Survival to hospital discharge, *n* (%)	14 (51.9)	19 (73.1)	21.2 (−4.2 to 46.6)	0.111
Survival at 6 months, *n* (%)	14 (51.9)	17 (65.4)	13.5 (−12.7 to 39.8)	0.318
CPC 1, 2 at discharge, *n* (%)	13 (48.1)	17 (65.4)	17.2 (−9.0 to 43.5)	0.206
CPC 1, 2 at 6 months, *n* (%)	13 (48.1)	16 (61.5)	13.4 (−13.2 to 39.9)	0.328

Values are expressed as numbers (percentages), medians (interquartile ranges), or means ± standard deviations. Other clinical outcomes are described in Supplementary Materials (Supplementary File S1). † The differences shown are expressed as percentage points or mean differences. Abbreviations: PS, Plasma Solution-A; CI, confidence interval; SBP, systolic blood pressure; ICU, intensive care unit; ED, emergency department; HR, heart rate; CVI, cumulative vasopressor index; AKI, acute kidney injury; MAKE30, major adverse kidney event within 30 days; CPC, cerebral performance category.

## Data Availability

The datasets used and/or analyzed during the current study are available from the corresponding author on reasonable request.

## Supplementary Materials

The following supporting information can be downloaded at: https://www.mdpi.com/article/10.3390/jcm12155040/s1, File S1: Study protocol; Table S1: Clinical outcomes of all patients who consented to the study; Table S2: Details of in-hospital treatment within 24 h in patients receiving saline versus Plasma Solution-A; Table S3: Laboratory results of patients receiving saline versus Plasma Solution-A; Table S4: Interval changes in the laboratory results; Table S5: Other clinical outcomes; Sensitivity analyses. References [6,8,13,14,15,16,31,37,38,39,40] are cited in the Supplementary Materials.
