# Supplementary material for: Saline versus Plasma Solution-A in Initial Resuscitation of Patients with Out-of-Hospital Cardiac Arrest: A Randomized Clinical Trial
Source: J Clin Med. 2023 Jul 31;12(15):5040. doi: 10.3390/jcm12155040 (PMC10420180; doi:10.3390/jcm12155040)
Supplement: Supplementary file 1 [file jcm-12-05040-s001.zip › jcm-2485575-supplementary.pdf]

Supplementary Material

Saline versus Plasma Solution-A in Initial Resuscitation of Patients with Out-of-Hospital Cardiac Arrest: A Randomized Clinical Trial

Jae-Hyug Woo, Yong Su Lim *, Jin Seong Cho, Hyuk Jun Yang, Jae Ho Jang, Jea Yeon Choi and Woo Sung Choi

Department of Emergency and Critical Care Medicine, Gachon University Gil Medical Center, Gachon University College of Medicine, Incheon 21565, Republic of Korea; emmetalkiller@gilhospital.com (J.-H.W.)

* Correspondence: yongem@gilhospital.com

Study protocol

Table S1. Clinical outcomes of all patients who consented to the study

Table S2. Details of in-hospital treatment within 24 h in patients receiving saline versus Plasma Solution-A

Table S3. Laboratory results of patients receiving saline versus Plasma Solution-A

Table S4. Interval changes in the laboratory results

Table S5. Other clinical outcomes

Sensitivity analyses

Study protocol

Trial Design

We conducted an investigator-initiated, pragmatic, randomized, unblinded, parallel-group clinical trial in which the use of a balanced crystalloid (Plasma Solution-A [PS]) was compared with the use of saline in intravenous (IV) fluid administration during cardiopulmonary resuscitation (CPR) and post-cardiac arrest care of non-traumatic out-of-hospital cardiac arrest (OHCA) patients admitted to a tertiary university hospital emergency department (ED) between April 1, 2019, and July 15, 2020 (clinical trial numbers KCT0003587, https://cris.nih.go.kr/cris/index.jsp).

No changes were made to the trial protocol after the trial commenced. The study protocol was approved by the Gachon University Gil Medical Center IRB (GCIRB2017-293). The IRB permitted an initial waiver of consent because it was not practical to obtain informed consent before initiating IV administration. We attempted to obtain consent from the surrogate as soon as possible after return of spontaneous circulation (ROSC). This trial complied with the principles of the Declaration of Helsinki. This manuscript follows the Consolidated Standards of Reporting Trials (CONSORT) guidelines.

Balanced Crystalloid (Plasma Solution-A [PS])

PS (HK inno.N Corp., Seoul, Republic of Korea) contains the same ingredients as Plasma-Lyte A and has the following electrolyte concentrations: sodium, 140 mEq/L; chloride (Cl^-), 98 mEq/L; acetate, 27 mEq/L; gluconate, 23 mEq/L; potassium, 5.0 mEq/L; magnesium, 3.0 mEq/L; pH, 7.4; and osmolality, 294 mOsm/L.

Study Population (Inclusion and Exclusion Criteria)

We consecutively screened patients aged ≥18 years who were admitted to our ED after non-traumatic OHCA irrespective of their initial cardiac rhythm. Patients were excluded if (1) they were presumed to be unsalvageable before ED visit (e.g., substantial rigor mortis, lividity, or cardiac arrest was presumed to have occurred a long time ago); (2) they were transferred from an outside institution; (3) they were pregnant; (4) they were on hemodialysis before ED visit; (5) the cardiac arrest cause (e.g., trauma, poisoning, alcoholic ketoacidosis, or diabetic ketoacidosis) was unclear; or (6) they had a do-not-resuscitate order or terminal cancer.

Sample Size Calculation

The sample size was calculated based on a previous randomized trial comparing the effect of a balanced crystalloid and saline on base excess (BE; difference in mean BE level=4.1 mmol/L; standard deviation of saline=4.6 mmol/L; and standard deviation of Plasma-Lyte A=3.9 mmol/L) [1]. The calculation was performed using the comparison of means method of MedCalc version 15.2.2 (MedCalc Software Ltd., Ostend, Belgium). Using a power of 90% at a two-sided α level of 0.05, a minimum of 24 participants were required in each group, resulting in 48 participants in total. Based on previous survival rates at 24 h obtained from our OHCA registry, we expected a minimum dropout rate of 70%.

Randomization

Participants were randomized 1:1 to receive either PS or saline for any IV administration of isotonic crystalloids. Randomization was achieved using permuted block randomization with a block size of 4. An author developed a randomization list using a web-based randomization program to generate trial IDs and treatment allocations before starting the trial. Blinding was not implemented.

Cardiopulmonary Resuscitation in Prehospital Settings

The Smart Advanced Life Support (SALS), an ongoing study, is being conducted near our hospital [2,3]. In the SALS, when OHCA occurs, emergency medical technicians who are directly supervised by medical directors (physicians) via real-time smartphone video calls perform advanced cardiovascular life support (ACLS) with IV fluid and some medications (such as epinephrine and amiodarone) in prehospital settings.

Trial Intervention and Follow-up

The trial interventions were performed for 24 h after the patient visited the ED. These interventions were continued in the intensive care unit (ICU) and ED. After receiving a notification that an OHCA patient was expected to visit our ED, the author designated the allocated crystalloid according to the randomization list. Upon arrival at the ED, all patients received only the allocated crystalloid while receiving ACLS. According to the allocation, the saline group received 0.9% sodium chloride when initial IV crystalloids were administered, whereas the PS group received PS. In cases where fluids were administered in prehospital settings, these were disconnected and removed immediately. We administered only the allocated crystalloid to the patient for the first 24 h in hospital. During the trial, the target mean arterial blood pressure

was above 65 mmHg and target urine output was above 0.5 mL/kg/h [4,5].

We restricted any other fluid administration, except for the allocated crystalloid, during the first 24 h. We did not mix sodium or potassium with the fluids being administered unless severe hyponatremia (<120 mEq/L) or severe hypokalemia (<2.5 mEq/L) occurred [5]. When using drugs essential for post-cardiac arrest care (e.g., vasopressors, sedatives, heparin, and nitroglycerin), they were administered at the maximum concentration possible after being mixed with the minimum volume of 5% dextrose. No other glucose-containing fluids were used unless hypoglycemia (<60 mg/dL) occurred. According to the written institutional protocol, hypoglycemia was treated with 50% or 10% dextrose. If hyperglycemia occurred, insulin was intravenously administered via bolus injection and continuous infusion with titration. We did not use hypertonic solutions, such as mannitol. Administration of sodium bicarbonate below 50 mEq was only permitted when metabolic acidosis persisted despite hemodynamic stability. In other cases, sodium bicarbonate administration was prohibited. Hemodialysis was initiated if acute kidney injury (AKI) with life-threatening conditions (e.g., hyperkalemia, pulmonary edema, or severe metabolic acidosis) occurred and did not respond to conservative treatments [6].

Upon arrival at the ED (0 h), blood samples were collected to determine serum electrolyte levels and perform a complete blood count, coagulation tests, liver and

renal function tests, and arterial blood gas analysis (ABGA). ABGA was subsequently performed at 30 min and 1, 2, 4, 6, 12, 18, and 24 h. We measured serum electrolyte and lactate levels at 6-h intervals for the first 24 h. Complete blood count and coagulation profiles were obtained at 12-h intervals for the first 24 h. Liver and renal function tests were performed once more at 24 h. Urinalysis was performed at 0 and 24 h.

These interventions were discontinued if (1) sustained ROSC was not achieved; (2) the patient died within 24 h; (3) quarantine was required because of coronavirus disease 2019; (4) consent was not obtained; (5) stroke was suspected to be the cause of OHCA, as indicated by computed tomography performed during the interventions; (6) a patient's family requested a do-not-resuscitate order and refused clinical examination, including laboratory tests; (7) hyperkalemia (>5.5 mEq/L), hypernatremia (>150 mEq/L), or hyperosmolality (>320 mOsm/kg) developed during the interventions [1]; (8) hemodialysis was required during the interventions; (9) an unassigned fluid was administered; or (10) a large amount of sodium bicarbonate (>50 mEq) was administered.

Data Collection

We obtained information concerning patient demographics, characteristics of

cardiac arrest, and amount and type of crystalloid administered in prehospital settings. We recorded the amount and type of IV fluids, blood products, and electrolytes administered for the first 24 h after the patient's presentation to the ED. We recorded the amount and type of drugs (e.g., diuretics, insulin, sodium bicarbonate, and vasopressors) administered. We collected laboratory results from blood and urine samples, and clinical data, including vital signs and total urine output. We monitored the patient's status for up to 6 months and recorded the neurologic and survival outcomes, determined by reviewing medical records or by telephone interviews with the patient or a primary caregiver.

Outcomes

The primary outcomes measured were changes in arterial pH, base excess (BE: primary endpoint), and bicarbonate ($HCO3^-$) and Cl^- levels within the first 24 h. The secondary outcomes were clinical outcomes, specifically AKI development within 72 h, major adverse kidney events within 30 days (MAKE30), stability of hemodynamic status within 24 h, and neurologic and survival outcomes at 6 months after OHCA (secondary endpoint: survival to hospital discharge).

Definitions

AKI was defined when any of the following criteria were met: an increase in serum creatinine (s-Cr) level by ≥0.3 mg/dL within 48 h; an increase in s-Cr level to ≥1.5 times baseline, which is known (the lowest s-Cr level between 12 months and 24 h before ED arrival) or is presumed according to the estimated baseline s-Cr level suggested by a previous report; or urine volume <0.5 mL/kg/h for 6 h [7,8].

MAKE30 was defined when any of the following criteria were met: death, new receipt of renal replacement therapy, or an increase in s-Cr level to ≥2 times the baseline s-Cr level [8].

To evaluate hemodynamic stability, we computed the cumulative vasopressor index (CVI) at 6 h, 12 h, 24 h, and the time of ICU admission [9–11].

Neurologic outcomes were assessed using the cerebral performance category (CPC) at 6 months after OHCA. The categories were defined as follows: CPC1, good performance; CPC2, moderate disability; CPC3, severe disability; CPC4, vegetative state; and CPC5, brain death or death. A good CPC (favorable outcomes) was defined as CPC1 or CPC2, while a poor CPC (unfavorable outcomes) was defined as CPC3 to CPC5.

References

1. Young JB, Utter GH, Schermer CR, et al. Saline versus Plasma-Lyte A in initial resuscitation of trauma patients: a randomized trial. Ann Surg 2014;259:255-62. https://doi.org/10.1097/SLA.0b013e318295feba.

2. Woo JH, Cho JS, Lee CA, et al. Survival and rearrest in out-of-hospital cardiac arrest patients with prehospital return of spontaneous circulation: a prospective multi-regional observational study. Prehosp Emerg Care 2021;25:59-66. https://doi.org/10.1080/10903127.2020.1733716.

3. Kim GW, Moon HJ, Lim H, et al. Effects of Smart Advanced Life Support protocol implementation including CPR coaching during out-of-hospital cardiac arrest. Am J Emerg Med 2022;56:211-7. https://doi.org/10.1016/j.ajem.2022.03.050.

4. Kim YM, Park KN, Choi SP, et al. Part 4. Post-cardiac arrest care: 2015 Korean Guidelines for Cardiopulmonary Resuscitation. Clin Exp Emerg Med 2016;3:S27-38. https://doi.org/10.15441/ceem.16.130.

5. Tintinalli JE, Stapczynski JS, Ma OJ, Yealy DM, Meckler GD, Cline D. Tintinalli's emergency medicine: a comprehensive study guide. New York: McGraw-Hill Education; 2016.

6. Kidney disease: improving global outcomes (KDIGO) acute kidney injury work group. Section 5: Dialysis interventions for treatment of AKI. Kidney Int Suppl 2012;2:89-115. https://doi.org/10.1038/kisup.2011.35.

7. Kidney disease: improving global outcomes (KDIGO) acute kidney injury work group. Section 2: AKI definition. Kidney Int Suppl 2012;2:19-36. https://doi.org/10.1038/kisup.2011.32.

8. Semler MW, Self WH, Wanderer JP, et al. Balanced crystalloids versus saline in critically ill adults. N Engl J Med 2018;378:829-39. https://doi.org/10.1056/NEJMoa1711584.

9. Song G, You Y, Jeong W, et al. Vasopressor requirement during targeted temperature management for out-of-hospital cardiac arrest caused by acute myocardial infarction without cardiogenic shock. Clin Exp Emerg Med 2016;3:20-6. https://doi.org/10.15441/ceem.15.090.

10. Trzeciak S, McCoy JV, Phillip Dellinger R, et al. Early increases in microcirculatory perfusion during protocol-directed resuscitation are associated with reduced multi-organ failure at 24 h in patients with sepsis. Intensive Care Med 2008;34:2210-7. https://doi.org/10.1007/s00134-008-1193-6.

11. Woo JH, Lim YS, Yang HJ, et al. The relationship between the decreased rate of initial blood glucose and neurologic outcomes in survivors of out-of-hospital cardiac arrest receiving therapeutic hypothermia. Neurocrit Care 2017;26:402-10. https://doi.org/10.1007/s12028-016-0353-8.

Table S1. Clinical outcomes of all patients who consented to the study

Variables	Saline group (n=183)	PS group (n=181)	p-value
ROSC (yes), n (%)	77 (42.1)	78 (43.1)	0.844
Sustained ROSC (yes), n (%)	73 (39.9)	75 (41.4)	0.764
Survival to hospital discharge, n (%)	27 (14.8)	28 (15.5)	0.849
Survival at 24 h, n (%)	56 (30.6)	54 (29.8)	0.873
Survival at 1 week, n (%)	40 (21.9)	36 (19.9)	0.644
Survival at 1 month, n (%)	27 (14.8)	26 (14.4)	0.916
Survival at 6 months, n (%)	27 (14.8)	23 (12.7)	0.571
CPC 1, 2 at discharge, n (%)	24 (13.1)	24 (13.3)	0.967
CPC 1, 2 at 1 month, n (%)	24 (13.1)	24 (13.3)	0.967
CPC 1, 2 at 6 months, n (%)	24 (13.1)	22 (12.2)	0.783

Values are expressed as numbers (percentages). Abbreviations: PS, Plasma Solution-A; ROSC, return of spontaneous circulation; CPC, cerebral performance category

Table S2. Details of in-hospital treatment within 24 h in patients receiving saline versus Plasma Solution-A

**Variables**	**Saline group (n=27)**	**PS group (n=26)**	**p-value**
Cumulative dose of diuretics administered (mg)	0.0 (0.0, 0.0)	0.0 (0.0, 0.0)	0.530
Cumulative dose of insulin administered (IU)	0.0 (0.0, 5.5)	0.0 (0.0, 7.0)	0.822
Cumulative dose of dextrose administered (g)	46.2±31.7	59.1±31.9	0.145
Cumulative dose of potassium replacement (mEq)	0.0 (0.0, 0.0)	0.0 (0.0, 0.0)	0.294
Cumulative dose of calcium replacement (mEq)	0.0 (0.0, 0.0)	0.0 (0.0, 0.0)	0.712
Cumulative dose of magnesium replacement (mEq)	0.0 (0.0, 0.0)	0.0 (0.0, 0.0)	0.321
Cumulative dose of phosphate replacement (mmol)	0.0 (0.0, 0.0)	N/C	0.326
Cumulative volume of PRBC transfusion (mL)	0.0 (0.0, 0.0)	0.0 (0.0, 0.0)	0.594
Cumulative volume of FFP transfusion (mL)	0.0 (0.0, 0.0)	0.0 (0.0, 0.0)	0.979

Values are expressed as medians (interquartile ranges) or means±standard deviations.

Abbreviations: PS, Plasma Solution-A; N/C, not calculated; PRBC, packed red blood cell; FFP, fresh frozen plasma

Table S3. Laboratory results of patients receiving saline versus Plasma Solution-A

Variables	Saline group (n=27)	PS group (n=26)	Absolute difference (95% CI)†	p-value
Arterial pH				
at baseline	7.08±0.19	7.11±0.18	0.03 (-0.08 to 0.13)	0.620*
at 30 min	7.23 (7.07, 7.28)	7.23 (7.15, 7.30)	0.03 (-0.07 to 0.13)	0.620*
at 1 h	7.27 (7.19, 7.32)	7.30 (7.23, 7.34)	0.04 (-0.04 to 0.13)	0.456*
at 2 h	7.30 (7.19, 7.36)	7.31 (7.27, 7.37)	0.05 (-0.02 to 0.13)	0.456*
at 4 h	7.31 (7.22, 7.37)	7.34 (7.28, 7.42)	0.05 (-0.01 to 0.11)	0.456*
at 6 h	7.29±0.11	7.35±0.12	0.07 (0.00 to 0.13)	0.108*
at 12 h	7.32 (7.25, 7.38)	7.36 (7.34, 7.40)	0.06 (0.00 to 0.12)	0.108*
at 18 h	7.30±0.10	7.37±0.10	0.06 (0.01 to 0.12)	0.108*

at 24 h	7.30±0.11	7.38±0.11	0.08 (0.02 to 0.14)	0.099*
Base excess				
at baseline (mmol/L)	-14.9 (-16.3, -9.3)	-12.8 (-15.4, -8.8)	-0.1 (-4.5 to 4.3)	0.595*
at 30 min (mmol/L)	-10.6±6.7	-9.1±7.2	1.5 (-2.6 to 5.5)	0.538*
at 1 h (mmol/L)	-9.3±6.9	-6.4±6.9	3.0 (-0.9 to 6.8)	0.167*
at 2 h (mmol/L)	-8.2±6.6	-3.6±6.0	4.6 (1.0 to 8.2)	0.032*
at 4 h (mmol/L)	-7.4±5.3	-3.8±5.8	3.6 (0.5 to 6.7)	0.036*
at 6 h (mmol/L)	-7.9±5.5	-4.1±4.9	3.7 (0.8 to 6.6)	0.032*
at 12 h (mmol/L)	-7.2 (-10.5, -4.9)	-4.7 (-6.4, -2.3)	4.1 (1.1 to 7.0)	0.009*
at 18 h (mmol/L)	-7.4±5.2	-2.9±5.1	4.5 (1.7 to 7.4)	0.009*
at 24 h (mmol/L)	-7.2±6.1	-3.4±5.5	3.9 (0.6 to 7.1)	0.036*

Arterial HCO_3^-				
at baseline (mmol/L)	14.1±4.5	14.8±5.4	0.7 (-2.2 to 3.7)	0.625*
at 30 min (mmol/L)	16.6±5.2	17.7±5.7	1.0 (-2.2 to 4.3)	0.583*
at 1 h (mmol/L)	17.7±5.4	19.9±5.5	2.2 (-0.9 to 5.2)	0.202*
at 2 h (mmol/L)	18.0±5.5	21.9±4.8	3.8 (0.9 to 6.8)	<u>0.034</u>*
at 4 h (mmol/L)	18.1±4.6	21.4±5.0	3.4 (0.7 to 6.1)	<u>0.034</u>*
at 6 h (mmol/L)	17.8±5.1	20.5±4.1	2.7 (0.1 to 5.3)	0.062*
at 12 h (mmol/L)	17.4±4.8	20.7±4.4	3.3 (0.7 to 5.8)	<u>0.034</u>*
at 18 h (mmol/L)	17.7±4.7	21.4±4.5	3.7 (1.2 to 6.2)	<u>0.034</u>*
at 24 h (mmol/L)	18.8 (15.0, 20.1)	20.2 (17.7, 22.8)	2.5 (-0.2 to 5.3)	0.062*
pCO_2				
at baseline (mmHg)	44.5 (36.0, 88.0)	49.0 (31.0, 84.0)	1.2 (-15.9 to 18.2)	0.756

at 30 min (mmHg)	44.0 (39.0, 52.5)	42.5 (37.0, 55.0)	3.3 (-7.4 to 14.1)	0.928
at 1 h (mmHg)	17.7±5.4	19.9±5.5	2.2 (-0.9 to 5.2)	0.157
at 2 h (mmHg)	37.0 (32.5, 47.0)	41.0 (34.6, 51.0)	3.3 (-4.4 to 11.1)	0.195
at 4 h (mmHg)	34.3 (30.0, 40.0)	39.0 (33.0, 47.0)	2.3 (-4.3 to 8.9)	0.184
at 6 h (mmHg)	32.2 (29.0, 39.9)	35.8 (31.2, 40.6)	-0.1 (-6.5 to 6.2)	0.389
at 12 h (mmHg)	32.1 (29.3, 37.2)	34.6 (29.0, 42.3)	1.5 (-3.9 to 6.8)	0.442
at 18 h (mmHg)	35.2±10.5	37.4±8.7	2.2 (-3.1 to 7.5)	0.410
at 24 h (mmHg)	36.1±8.9	34.5±7.1	-1.6 (-6.1 to 2.9)	0.490
Arterial lactate				
at baseline (mmol/L)	10.0±3.7	10.6±2.9	0.5 (-1.3 to 2.4)	0.553
at 30 min (mmol/L)	7.0±3.6	8.1±3.1	1.1 (-0.8 to 3.0)	0.255
at 1 h (mmol/L)	5.7 (3.1, 7.0)	5.7 (3.6, 8.3)	0.1 (-1.7 to 1.8)	0.600

at 2 h (mmol/L)	3.0 (2.0, 5.1)	3.3 (2.4, 4.7)	-0.2 (-1.8 to 1.3)	0.661
at 4 h (mmol/L)	3.2±2.0	3.3±2.0	0.1 (-1.0 to 1.2)	0.859
at 6 h (mmol/L)	2.2 (1.6, 3.8)	2.8 (1.5, 3.5)	0.0 (-1.2 to 1.2)	0.819
at 12 h (mmol/L)	2.5 (1.5, 3.4)	2.5 (1.8, 4.7)	0.6 (-0.4 to 1.6)	0.366
at 18 h (mmol/L)	2.6±2.0	2.7±1.8	0.1 (-1.0 to 1.1)	0.880
at 24 h (mmol/L)	1.5 (1.1, 2.8)	2.5 (1.2, 3.9)	1.0 (-0.4 to 2.5)	0.180
Arterial ionized magnesium				
at baseline (mmol/L)	N/D	N/D	N/C	
at 30 min (mmol/L)	N/D	N/D	N/C	
at 1 h (mmol/L)	N/D	N/D	N/C	
at 2 h (mmol/L)	0.5±0.2	0.6±0.1	0.1 (-0.2 to 0.3)	0.552
at 4 h (mmol/L)	0.5±0.1	0.5±0.1	0.0 (-0.1 to 0.1)	0.604

at 6 h (mmol/L)	0.5±0.2	0.5±0.1	0.0 (-0.1 to 0.1)	0.868
at 12 h (mmol/L)	0.5±0.1	0.5±0.1	0.0 (-0.1 to 0.1)	0.654
at 18 h (mmol/L)	0.5±0.1	0.5±0.1	0.0 (0.0 to 0.1)	0.173
at 24 h (mmol/L)	0.5 (0.4, 0.6)	0.5 (0.5, 0.6)	0.0 (0.0 to 0.1)	0.844
Serum sodium				
at baseline (mEq/L)	139.0 (137.0, 140.0)	138.0 (136.0, 140.0)	0.0 (-2.2 to 2.2)	0.707
at 6 h (mEq/L)	138.0 (136.0, 139.0)	137.0 (135.0, 138.0)	-0.9 (-2.9 to 1.2)	0.258
at 12 h (mEq/L)	138.0 (136.5, 139.0)	135.5 (134.0, 138.0)	-1.9 (-3.9 to 0.1)	<u>0.027</u>
at 18 h (mEq/L)	139.0 (136.0, 140.0)	135.5 (134.0, 137.0)	-3.2 (-5.4 to -1.0)	<u>0.003</u>
at 24 h (mEq/L)	139.0 (137.0, 140.5)	136.0 (133.0, 138.0)	-3.0 (-5.2 to -0.8)	<u>0.003</u>
Serum potassium				
at baseline (mEq/L)	4.0±1.0	3.9±0.8	-0.2 (-0.7 to 0.3)	0.510

at 6 h (mEq/L)	4.0±0.8	3.8±0.6	-0.3 (-0.7 to 0.1)	0.141
at 12 h (mEq/L)	3.9±0.8	3.9±0.8	-0.1 (-0.5 to 0.4)	0.786
at 18 h (mEq/L)	4.2±1.0	3.7±0.6	-0.5 (-0.9 to 0.0)	<u>0.033</u>
at 24 h (mEq/L)	4.1±0.9	3.7±0.6	-0.4 (-0.9 to 0.0)	0.054
Serum chloride				
at baseline (mEq/L)	101.9±6.3	101.1±5.1	-0.8 (-4.0 to 2.3)	0.593*
at 6 h (mEq/L)	105.9±6.3	100.7±5.0	-5.2 (-8.4 to -2.1)	<u>0.003</u>*
at 12 h (mEq/L)	106.9±7.0	100.4±4.2	-6.5 (-9.7 to -3.3)	<u><0.001</u>*
at 18 h (mEq/L)	108.3±6.4	99.7±4.1	-8.7 (-11.6 to -5.7)	<u><0.001</u>*
at 24 h (mEq/L)	108.6±5.7	99.8±4.8	-8.8 (-11.7 to -5.9)	<u><0.001</u>*
Serum measured osmolality				
at baseline (mOsm/kg)	298.0 (293.5, 309.5)	301.5 (293.0, 305.0)	1.6 (-9.5 to 12.7)	0.894

at 6 h (mOsm/kg)	293.8±10.9	291.1±12.1	-2.7 (-9.1 to 3.7)	0.397
at 12 h (mOsm/kg)	291.0 (285.5, 299.5)	285.5 (281.0, 293.0)	21.2 (-33.2 to 75.6)	0.071
at 18 h (mOsm/kg)	290.0 (285.0, 298.0)	285.0 (279.0, 290.0)	-5.3 (-12.8 to 2.1)	<u>0.015</u>
at 24 h (mOsm/kg)	291.0±10.7	283.8±11.0	-7.2 (-13.2 to -1.3)	<u>0.019</u>
Serum calcium				
at baseline (mg/dL)	8.4±0.9	8.3±0.6	-0.1 (-0.5 to 0.3)	0.579
at 6 h (mg/dL)	7.6±0.6	7.7±0.5	0.1 (-0.2 to 0.4)	0.360
at 12 h (mg/dL)	7.6 (7.0, 8.0)	7.4 (6.8, 8.0)	-0.3 (-0.9 to 0.3)	0.606
at 18 h (mg/dL)	7.4±0.8	7.4±0.7	0.1 (-0.3 to 0.5)	0.660
at 24 h (mg/dL)	7.5 (6.7, 7.8)	7.3 (6.9, 7.8)	0.2 (-0.2 to 0.6)	0.593
Serum ionized calcium				
at baseline (mmol/L)	1.2±0.1	1.2±0.1	0.0 (-0.1 to 0.0)	0.473

at 6 h (mmol/L)	1.1±0.1	1.1±0.1	0.0 (-0.1 to 0.0)	0.033
at 12 h (mmol/L)	1.1±0.1	1.0±0.1	-0.1 (-0.1 to 0.0)	0.092
at 18 h (mmol/L)	1.1±0.1	1.1±0.1	0.0 (-0.1 to 0.0)	0.164
at 24 h (mmol/L)	1.1±0.1	1.1±0.1	0.0 (-0.1 to 0.0)	0.532
Serum phosphate				
at baseline (mg/dL)	6.9±2.4	6.3±1.9	-0.5 (-1.7 to 0.6)	0.364
at 6 h (mg/dL)	3.7±1.2	3.2±1.5	-0.5 (-1.3 to 0.3)	0.193
at 12 h (mg/dL)	3.5 (2.8, 4.1)	2.7 (2.5, 3.5)	-0.4 (-1.0 to 0.3)	0.067
at 18 h (mg/dL)	3.7±1.2	3.2±0.9	-0.5 (-1.1 to 0.1)	0.091
at 24 h (mg/dL)	3.9±1.6	3.2±1.3	-0.6 (-1.4 to 0.1)	0.108
Serum magnesium				
at baseline (mg/dL)	2.4 (2.1, 2.5)	2.2 (2.0, 2.4)	0.0 (-0.2 to 0.2)	0.403

at 6 h (mg/dL)	1.9±0.2	1.9±0.3	0.1 (-0.1 to 0.2)	0.486
at 12 h (mg/dL)	1.8 (1.7, 2.0)	1.8 (1.6, 1.9)	0.0 (-0.2 to 0.2)	0.879
at 18 h (mg/dL)	1.8±0.3	1.9±0.3	0.2 (0.0 to 0.4)	<u>0.029</u>
at 24 h (mg/dL)	1.7±0.3	2.0±0.3	0.3 (0.1 to 0.5)	<u>0.001</u>
Venous lactate				
at baseline (mg/dL)	98.0±49.8	88.4±33.2	-9.6 (-33.0 to 13.9)	0.417
at 6 h (mg/dL)	32.0±20.6	33.7±19.8	1.7 (-9.7 to 13.0)	0.768
at 12 h (mg/dL)	30.6 (20.6, 35.7)	26.4 (20.2, 44.0)	4.7 (-4.3 to 13.7)	0.705
at 18 h (mg/dL)	22.3 (17.5, 32.6)	23.8 (17.2, 43.7)	5.5 (-6.1 to 17.1)	0.510
at 24 h (mg/dL)	20.0 (12.5, 37.8)	19.6 (12.6, 37.7)	4.0 (-9.2 to 17.2)	0.847
Hemoglobin				
at baseline (g/dL)	13.0 (10.8, 13.9)	13.9 (10.8, 14.7)	0.8 (-0.7 to 2.2)	0.336

at 12 h (g/dL)	12.9±2.5	13.3±2.7	0.4 (-1.0 to 1.9)	0.563
at 24 h (g/dL)	12.3±2.9	13.1±2.7	0.8 (-0.8 to 2.4)	0.315
Hematocrit				
at baseline (%)	40.7 (34.4, 43.4)	42.3 (36.5, 45.5)	2.8 (-1.4 to 7.0)	0.255
at 12 h (%)	38.2±6.5	39.3±7.8	1.1 (-2.8 to 5.1)	0.565
at 24 h (%)	36.3±8.0	38.4±7.7	2.1 (-2.3 to 6.4)	0.344
RBC				
at baseline ($10^6/mm^3$)	3.9±1.0	4.2±0.7	0.3 (-0.2 to 0.8)	0.195
at 12 h ($10^6/mm^3$)	4.2±0.8	4.4±0.9	0.2 (-0.3 to 0.7)	0.415
at 24 h ($10^6/mm^3$)	4.0±1.0	4.3±0.8	0.3 (-0.2 to 0.8)	0.209
WBC				
at baseline ($10^3/mm^3$)	12.9±4.7	12.5±3.8	-0.4 (-2.8 to 2.0)	0.735

at 12 h ($10^3/mm^3$)	15.9 (12.2, 20.0)	10.9 (9.6, 13.4)	-4.6 (-8.6 to -0.7)	0.016
at 24 h ($10^3/mm^3$)	13.8 (11.3, 17.6)	12.3 (8.7, 13.9)	-1.8 (-5.2 to 1.6)	0.122
Platelets				
at baseline ($10^3/mm^3$)	229.0 (155.0, 301.0)	234.0 (199.0, 274.0)	10.0 (-38.8 to 58.8)	0.894
at 12 h ($10^3/mm^3$)	223.2±82.2	226.9±81.1	3.7 (-41.3 to 48.7)	0.870
at 24 h ($10^3/mm^3$)	191.5±67.0	206.9±93.8	15.4 (-29.4 to 60.2)	0.493
C-reactive protein				
at baseline (mg/dL)	0.1 (0.1, 0.6)	0.1 (0.0, 0.7)	-1.5 (-3.1 to 0.1)	0.460
at 12 h (mg/dL)	1.6 (1.0, 2.3)	1.5 (0.6, 2.3)	-1.8 (-3.9 to 0.2)	0.482
at 24 h (mg/dL)	5.8 (3.2, 9.8)	3.9 (3.0, 10.0)	-1.2 (-3.9 to 1.5)	0.444
PT				
at baseline (s)	12.2 (11.2, 14.3)	11.9 (11.0, 12.7)	-2.4 (-6.3 to 1.5)	0.290

at 12 h (s)	13.3 (12.2, 16.4)	12.8 (11.7, 13.9)	-1.9 (-4.8 to 1.0)	0.219
at 24 h (s)	13.1 (12.4, 14.9)	12.8 (11.7, 14.4)	-2.1 (-6.1 to 1.9)	0.449
PT/INR				
at baseline	1.1 (1.0, 1.3)	1.1 (1.0, 1.1)	-0.8 (-2.4 to 0.8)	0.266
at 12 h	1.2 (1.1, 1.4)	1.1 (1.0, 1.2)	-0.2 (-0.4 to 0.1)	0.229
at 24 h	1.2 (1.1, 1.3)	1.1 (1.0, 1.3)	-0.3 (-0.7 to 0.2)	0.444
PTT				
at baseline (s)	33.9 (30.0, 41.4)	30.7 (27.7, 36.3)	-4.7 (-14.2 to 4.9)	0.230
at 12 h (s)	34.9 (30.9, 64.7)	34.6 (30.2, 55.2)	-4.5 (-19.3 to 10.4)	0.510
at 24 h (s)	40.0 (31.5, 59.1)	41.9 (30.6, 88.5)	10.6 (-6.5 to 27.6)	0.624
FDP				
at baseline (μg/mL)	46.0 (17.1, 89.7)	21.8 (4.4, 53.9)	-17.9 (-40.4 to 4.6)	0.137

at 12 h (μg/mL)	16.7 (6.0, 39.5)	11.8 (4.1, 21.1)	-10.7 (-32.2 to 10.7)	0.230
at 24 h (μg/mL)	9.8 (4.9, 26.0)	5.3 (3.0, 13.7)	-8.4 (-26.5 to 9.7)	0.081
Fibrinogen				
at baseline (mg/dL)	338.7±160.4	358.5±104.9	19.8 (-55.2 to 94.9)	0.598
at 12 h (mg/dL)	395.5±159.3	403.3±104.8	7.8 (-67.3 to 82.9)	0.836
at 24 h (mg/dL)	421.0 (392.0, 471.5)	509.0 (418.0, 570.0)	10.5 (-65.6 to 86.6)	0.160
Total bilirubin				
at baseline (mg/dL)	0.6±0.2	0.7±0.3	0.0 (-0.1 to 0.2)	0.906
at 24 h (mg/dL)	0.8±0.4	1.2±0.6	0.4 (0.1 to 0.6)	<u>0.013</u>
AST				
at baseline (U/L)	121.0 (81.0, 210.0)	95.5 (42.0, 127.0)	-85.9 (-189.6 to 17.8)	<u>0.047</u>
at 24 h (U/L)	116.0 (59.5, 251.5)	65.5 (44.0, 105.0)	-164.9 (-371.0 to 41.2)	0.100

ALT				
at baseline (U/L)	90.0 (51.5, 191.0)	52.0 (36.0, 131.0)	-81.0 (-185.0 to 23.1)	0.077
at 24 h (U/L)	137.0 (51.5, 275.5)	65.5 (35.0, 118.0)	-198.3 (-376.2 to -20.4)	0.026
Serum protein				
at baseline (g/dL)	6.2±0.8	6.3±0.8	0.1 (-0.3 to 0.5)	0.666
at 24 h (g/dL)	5.4±0.9	5.6±1.0	0.3 (-0.2 to 0.8)	0.265
Serum albumin				
at baseline (g/dL)	3.7 (3.4, 4.1)	3.9 (3.4, 4.1)	0.0 (-0.3 to 0.3)	0.768
at 24 h (g/dL)	3.3±0.6	3.4±0.7	0.1 (-0.3 to 0.5)	0.571
Serum BUN				
at baseline (mg/dL)	17.1±9.1	15.1±6.8	-2.0 (-6.4 to 2.5)	0.376
at 24 h (mg/dL)	19.2 (14.9, 27.0)	17.9 (11.6, 21.5)	-5.9 (-12.3 to 0.6)	0.194

Serum creatinine				
at baseline (mg/dL)	1.2 (0.9, 1.3)	1.1 (0.9, 1.3)	-0.2 (-0.4 to 0.0)	0.434
at 24 h (mg/dL)	0.7 (0.6, 1.0)	0.7 (0.5, 0.9)	-0.2 (-0.6 to 0.1)	0.413
Serum amylase				
at baseline (U/L)	62.0 (50.5, 98.0)	72.5 (58.0, 100.0)	5.1 (-12.6 to 22.7)	0.278
at 24 h (U/L)	88.0 (43.5, 287.0)	126.5 (66.0, 213.0)	18.4 (-227.0 to 263.8)	0.359
Serum ammonia				
at baseline (μg/dL)	133.0 (90.0, 246.5)	117.0 (72.0, 211.0)	-37.8 (-110.6 to 35.1)	0.499
at 24 h (μg/dL)	59.3±36.7	55.0±30.4	-4.2 (-22.8 to 14.4)	0.651
Serum creatine phosphokinase				
at baseline (U/L)	142.0 (105.5, 176.0)	140.5 (95.0, 194.0)	-332.5 (-917.9 to 252.9)	0.755
at 24 h (U/L)	521.0 (315.5, 925.5)	366.5 (181.0, 1075.0)	221.6 (-654.4 to 1097.5)	0.545

Serum lactate dehydrogenase				
at baseline (U/L)	665.0 (567.5, 865.5)	666.5 (522.0, 860.0)	-67.5 (-309.7 to 174.7)	0.644
at 24 h (U/L)	786.0 (488.0, 1246.0)	670.5 (469.0, 997.0)	-204.8 (-772.9 to 363.3)	0.618
Serum calculated osmolality				
at baseline (mOsm/kg)	296.6 (292.6, 301.3)	295.6 (292.2, 300.0)	-0.5 (-5.9 to 4.9)	0.606
at 24 h (mOsm/kg)	293.6 (289.1, 298.7)	288.1 (285.3, 291.9)	-6.1 (-11.2 to -0.9)	<u>0.009</u>
Urine specific gravity				
at baseline	1.01 (1.01, 1.01)	1.01 (1.01, 1.01)	0.00 (0.00 to 0.01)	0.094
at 24 h	1.02 (1.01, 1.03)	1.02 (1.01, 1.03)	0.00 (-0.01 to 0.01)	0.642
Urine pH				
at baseline	6.50 (5.50, 6.75)	6.00 (5.50, 6.50)	-0.10 (-0.53 to 0.33)	0.626

at 24 h	5.50 (5.00, 5.50)	5.50 (5.00, 6.50)	0.22 (-0.24 to 0.68)	0.644

Values are expressed as medians (interquartile ranges) or means±standard deviations. * indicates corrected p-values using the Benjamini–Hochberg procedure because multiple comparisons were conducted. †The differences shown are expressed as percentage points or mean differences. Abbreviations: PS, Plasma Solution-A; CI, confidence interval; HCO_3^-, bicarbonate; pCO_2, partial pressure of carbon dioxide; N/D, not detected in all cases; N/C, not calculated; RBC, red blood cell; WBC, white blood cell; PT, prothrombin time; PT/INR, prothrombin time/international normalized ratio; PTT, partial thromboplastin time; FDP, fibrinogen degradation production; AST, aspartate aminotransferase; ALT, alanine transaminase; BUN, blood urea nitrogen

Table S4. Interval changes in the laboratory results

Variables	Saline group (n=27)	PS group (n=26)	Absolute difference (95% CI)†	p-value
Change in base excess				
(0–30 min, mmol/L)	2.8 (0.8, 4.8)	3.6 (0.6, 5.7)	2.1 (-1.6 to 5.8)	0.646
(30 min–2 h, mmol/L)	3.1±3.2	5.1±3.3	2.0 (0.1 to 4.0)	0.044
(2–6 h, mmol/L)	0.3±4.3	-0.6±3.6	-0.9 (-3.2 to 1.4)	0.450
(6–12 h, mmol/L)	0.2±1.6	0.5±2.5	0.4 (-0.8 to 1.5)	0.545
(12–24 h, mmol/L)	0.3±2.8	0.4±2.6	0.1 (-1.4 to 1.6)	0.878
Change in arterial HCO_3^-				
(0–30 min, mmol/L)	2.4 (1.0, 3.6)	2.7 (1.1, 4.8)	0.7 (-0.9 to 2.3)	0.524
(30 min–2 h, mmol/L)	1.9±2.8	3.8±2.4	1.9 (0.3 to 3.5)	0.024

(2–6 h, mmol/L)	-0.2±4.7	-1.2±3.3	-1.0 (-3.4 to 1.3)	0.371
(6–12 h, mmol/L)	-0.3±2.5	0.3±2.7	0.6 (-0.8 to 2.1)	0.399
(12–24 h, mmol/L)	0.3±2.3	-0.1±2.4	-0.4 (-1.7 to 0.9)	0.543
Change in arterial pH				
(0–30 min)	0.05 (-0.02, 0.17)	0.04 (-0.01, 0.18)	0.02 (-0.06 to 0.10)	0.808
(30 min–2 h)	0.10±0.10	0.11±0.12	0.01 (-0.05 to 0.07)	0.761
(2–6 h)	0.01 (-0.02, 0.05)	0.04 (-0.01, 0.07)	0.02 (-0.03 to 0.07)	0.345
(6–12 h)	0.01±0.07	0.00±0.08	-0.01 (-0.05 to 0.04)	0.784
(12–24 h)	-0.01±0.09	0.02±0.08	0.02 (-0.03 to 0.07)	0.338
Change in serum chloride				
(0–6 h, mEq/L)	4.0±4.2	-0.4±2.8	-4.4 (-6.4 to -2.4)	<0.001
(6–12 h, mEq/L)	0.0 (-0.5, 3.0)	0.0 (-2.0, 2.0)	-1.3 (-2.6 to 0.0)	0.100

(12–18 h, mEq/L)	2.0 (0.0, 3.0)	0.0 (-2.0, 1.0)	-2.2 (-3.5 to -0.8)	0.003
(18–24 h, mEq/L)	0.3±2.0	0.1±2.6	-0.1 (-1.4 to 1.1)	0.821
Increase in serum chloride				
(0–6 h, yes), n (%)	21 (77.8)	9 (34.6)	-43.2 (-67.3 to -19.1)	<0.001
(6–12 h, yes), n (%)	13 (48.1)	10 (38.5)	-9.7 (-36.2 to 16.9)	0.450
(12–18 h, yes), n (%)	16 (59.3)	8 (30.8)	-28.5 (-54.2 to -2.8)	0.083
(18–24 h, yes), n (%)	12 (44.4)	11 (42.3)	-2.1 (-28.8 to 24.6)	0.732
Development of hyperchloremia				
at 6 h (yes), n (%)	6 (22.2)	0 (0.0)	N/C	0.023
at 12 h (yes), n (%)	9 (33.3)	0 (0.0)	N/C	0.002
at 18 h (yes), n (%)	11 (40.7)	0 (0.0)	N/C	<0.001
at 24 h (yes), n (%)	10 (37.0)	0 (0.0)	N/C	0.001

Development of hypernatremia				
at 6 h (yes), n (%)	0 (0.0)	0 (0.0)	N/C	N/C
at 12 h (yes), n (%)	0 (0.0)	0 (0.0)	N/C	N/C
at 18 h (yes), n (%)	1 (3.7)	0 (0.0)	N/C	1.000
at 24 h (yes), n (%)	0 (0.0)	0 (0.0)	N/C	N/C
Development of hyperosmolar state				
at 6 h (yes), n (%)	2 (7.4)	1 (3.8)	-3.6 (-15.9 to 8.8)	1.000
at 12 h (yes), n (%)	2 (7.4)	1 (3.8)	-3.6 (-15.9 to 8.8)	1.000
at 18 h (yes), n (%)	1 (3.7)	1 (3.8)	0.1 (-10.1 to 10.4)	1.000
at 24 h (yes), n (%)	2 (7.4)	1 (3.8)	-3.6 (-15.9 to 8.8)	1.000
Development of hypoosmolar state				
at 6 h (yes), n (%)	7 (25.9)	9 (34.6)	8.7 (-16.0 to 33.3)	0.491

at 12 h (yes), n (%)	11 (40.7)	14 (53.8)	13.1 (-13.6 to 39.8)	0.339
at 18 h (yes), n (%)	10 (37.0)	17 (65.4)	28.4 (2.5 to 54.2)	0.039
at 24 h (yes), n (%)	13 (48.1)	19 (73.1)	24.9 (-0.5 to 50.3)	0.064

Values are expressed as numbers (percentages), medians (interquartile ranges), or means±standard deviations. †The differences shown are expressed as percentage points or mean differences. Abbreviations: PS, Plasma Solution-A; CI, confidence interval; HCO_3^-, bicarbonate; hyperchloremia, serum chloride level >110 mEq/L; N/C, not calculated; hypernatremia, serum sodium level >145 mEq/L; hyperosmolar state, serum osmolality >308 mOsm/kg; hypoosmolar state, serum osmolality <289 mOsm/kg

Table S5. Other clinical outcomes

Variables	Saline group (n=27)	PS group (n=26)	Absolute difference (95% CI)†	p-value
DBP at ICU admission (mmHg)	79.1±28.2	76.3±24.2	-2.8 (-17.3 to 11.7)	0.700
at 6 h after ED visit (mmHg)	70.1±17.9	76.5±22.8	6.4 (-4.9 to 17.7)	0.261
at 12 h after ED visit (mmHg)	74.1±18.8	75.2±18.2	1.1 (-9.1 to 11.3)	0.832
at 24 h after ED visit (mmHg)	73.1±16.3	74.3±11.8	1.2 (-6.6 to 9.1)	0.754
Highest DBP within 24 h (mmHg)	102.3±23.3	102.7±25.7	0.4 (-13.1 to 13.9)	0.953
Lowest DBP within 24 h (mmHg)	48.4±11.7	50.0±12.8	1.6 (-5.2 to 8.3)	0.647
Survival at 1 week, n (%)	20 (74.1)	20 (76.9)	N/C	0.810
Survival at 1 month, n (%)	14 (51.9)	19 (73.1)	21.2 (-4.2 to 46.6)	0.111
CPC 1, 2 at 1 month, n (%)	13 (48.1)	17 (65.4)	17.2 (-9.0 to 43.5)	0.206

Values are expressed as numbers (percentages), medians (interquartile ranges), or means±standard deviations. †The differences shown are expressed as percentage points or mean differences. Abbreviations: PS, Plasma Solution-A; CI, confidence interval; DBP, diastolic blood pressure; ICU, intensive care unit; ED, emergency department; N/C, not calculated; CPC, cerebral performance category

Sensitivity analyses

Increase in base excess within the first 24 h (primary endpoint)

Increase in base excess (0–24 h, mmol/L)	Saline group (n=27)	PS group (n=26)	Absolute difference (95% CI)†	p-value
Means±standard deviations, unadjusted	3.9±7.9	9.0±5.4	5.2 (1.2–9.2)	0.012
Means (standard errors), adjusted*	3.8 (1.4)	9.1 (1.4)	5.3 (1.2–9.4)	0.012

†The differences shown are expressed as mean differences. *Analysis of covariance (ANCOVA) was performed to analyze the increase in base excess within the first 24 h. The covariates included in this analysis were age, sex, initial rhythm (shockable), cardiac etiology, interval from collapse to basic life support, and bystander cardiopulmonary resuscitation. Abbreviations: PS, Plasma Solution-A; CI, confidence interval

Multivariable analysis of survival to hospital discharge (secondary endpoint)

Analysis	n	Adjusted odds ratio	95% CI	p-value
All patients	364	1.51	0.67–3.41	0.323
Excluding patients without ROSC	155	1.81	0.69–4.73	0.229
Excluding patients who could not adhere to the trial protocol	53	20.29	0.97–426.05	0.053

A binary logistic regression analysis was performed for survival to hospital discharge. The covariates included in these analyses were age, sex, initial rhythm (shockable), cardiac etiology, interval from collapse to basic life support, and bystander cardiopulmonary resuscitation. Each odds ratio was calculated using saline administration as a reference. Abbreviations: CI, confidence interval; ROSC, return of spontaneous circulation
